# Synthetic control devices for gene regulation in *Penicillium chrysogenum*

**DOI:** 10.1186/s12934-019-1253-3

**Published:** 2019-11-18

**Authors:** László Mózsik, Zsófia Büttel, Roel A. L. Bovenberg, Arnold J. M. Driessen, Yvonne Nygård

**Affiliations:** 1 Molecular Microbiology, Groningen Biomolecular Sciences and Biotechnology Institute, University of Groningen, Nijenborgh 7, 9747 AG Groningen, The Netherlands; 2 DSM Biotechnology Center, Alexander Fleminglaan 1, 2613 AX Delft, The Netherlands; 3 Synthetic Biology and Cell Engineering, Groningen Biomolecular Sciences and Biotechnology Institute, University of Groningen, Nijenborgh 7, 9747 AG Groningen, The Netherlands; 4 Division of Industrial Biotechnology, Department of Biology and Biological Engineering, Chalmers University of Technology, Kemivägen 10, 412 96 Gothenburg, Sweden

**Keywords:** Synthetic expression system, Gene regulation, *Penicillium chrysogenum*, Synthetic gene cluster, Secondary metabolite production, Hybrid transcription factor

## Abstract

**Background:**

Orthogonal, synthetic control devices were developed for *Penicillium chrysogenum*, a model filamentous fungus and industrially relevant cell factory. In the synthetic transcription factor, the QF DNA-binding domain of the transcription factor of the quinic acid gene cluster of *Neurospora crassa* is fused to the VP16 activation domain. This synthetic transcription factor controls the expression of genes under a synthetic promoter containing quinic acid upstream activating sequence (QUAS) elements, where it binds. A gene cluster may demand an expression tuned individually for each gene, which is a great advantage provided by this system.

**Results:**

The control devices were characterized with respect to three of their main components: expression of the synthetic transcription factors, upstream activating sequences, and the affinity of the DNA binding domain of the transcription factor to the upstream activating domain. This resulted in synthetic expression devices, with an expression ranging from hardly detectable to a level similar to that of highest expressed native genes. The versatility of the control device was demonstrated by fluorescent reporters and its application was confirmed by synthetically controlling the production of penicillin.

**Conclusions:**

The characterization of the control devices in microbioreactors, proved to give excellent indications for how the devices function in production strains and conditions. We anticipate that these well-characterized and robustly performing control devices can be widely applied for the production of secondary metabolites and other compounds in filamentous fungi.
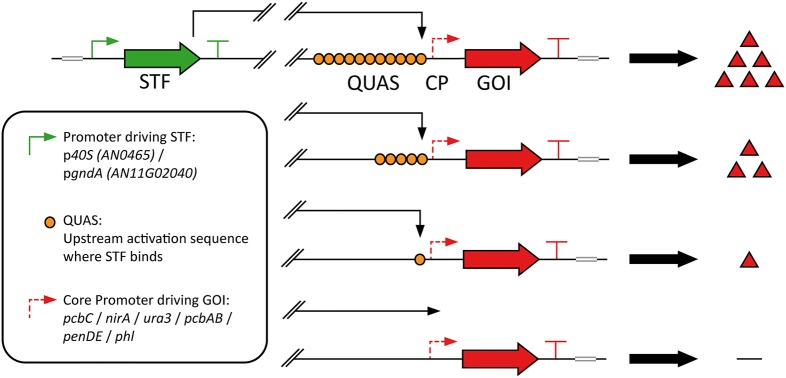

## Background

Synthetic biology has revolutionized metabolic engineering and takes the exploitation of industrial microorganism to a new level by enabling fine-tuning of gene expression and control of entire pathways. Recent advances such as CRISPR/Cas9 technologies accelerate strain construction and enable complex pathway engineering of also more challenging hosts [1]. The metabolic diversity and the wide range of ecological niches that fungi inhabit gives them a great potential as sources of novel enzymes and the use of fungi in white and red biotechnology is well established [2]. Thus, there is a great demand for synthetic biology tools for fungal cell factories.

Filamentous fungi such as *Penicillium chrysogenum* produce a variety of interesting secondary metabolites (SMs), compounds that are not essential for growth, but typically possess bioactivities that are of great value to medicine, agriculture and manufacturing. *P. chrysogenum* is well-known to produce β-lactam antibiotics, but it naturally produces a wide variety of SMs, and it has also been engineered to produce heterologous compounds [3]. Still, many SM clusters are not expressed under laboratory conditions and may need to be activated or heterologously expressed before the SMs can be obtained [4]. A further challenge is that many SMs genes are carbon catabolite repressed [5]. The number of sequenced filamentous fungi has lately seen a great increase, highlighting the need for orthogonal tools to explore the enormous potential of new SM biosynthetic clusters and their respective natural products. So far, no orthogonal expression systems have been used for activation of entire gene clusters in fungi and the challenge in expression of silent SM clusters forms a bottleneck in exploring the diversity of natural SM products. Therefore, the development of efficient expression devices is of great importance.

A number of promoters have been characterized for *P. chrysogenum* [6] and a couple of expression systems for protein production and secretion with *Penicillium* have been reported [7, 8]. The use of a promoter and its transcription factor from a native SM cluster for the production of high amounts of heterologous SMs, has been demonstrated in *Fusarium heterosporum* [9]. Similarly, a heterologous fungal expression system based on regulatory elements of the terrein gene cluster of *Aspergillus terreus* was demonstrated in *A. niger* [10]. Synthetic gene expression systems consisting of heterologous and hybrid transcription factors (TFs) composed of different DNA-binding and activation domains have previously been demonstrated in *Aspergilli* [11–14], *Ustilago maydis* [15] and *Trichoderma reesei* [13]. The systems developed were induced by doxycycline/tetracycline [11, 12, 15, 16] or estradiol [17] and while widely useful for proof-of-concept studies, the need of an inducer provides a physiological complication [18] and potential commercial hindrance [19]. The recent synthetic expression systems developed by Rantasalo et al. [13], where the transcription factor was expressed using a core promoter (CP) instead of a full-length promoter. These systems were shown to be functional in several yeasts and two filamentous fungi.

An increasing number of promoter libraries have been designed for prokaryotes and yeast, by random sequence modifications or by rational approaches, including introduction of various upstream activating sequence (UAS) elements [20] or evaluating different CPs [19, 21]. TFs conferring specific activation/repression mechanisms interact with designated UAS elements, but a CP (sometimes called minimal promoter) sequence is required to recruit general transcription factors and the RNA polymerase II for transcription initiation (reviewed by Juven-Gershon and Kadonaga [22]). The CP is the minimal portion of the promoter required to initiate transcription, typically containing the site for start of transcription, the polymerase binding site and general transcription factor binding sites, such as the TATA and CCAAT boxes and the initiator element [22]. These CP elements are found in some but not all promoters and the sequence—function relationship of these elements remains unclear. CPs of the *nirA* [17] and *gaaC* [13] genes of *A. niger* and of the *gpdA* [12] gene of *A. nidulans*, as well as the *ura3* gene of *Saccharomyces cerevisiae* [17] have been demonstrated to function in *Aspergilli*. The transcription start sites have been described for the penicillin biosynthesis gene cluster of *P. chrysogenum* [23] but so far there are no CPs demonstrated in this fungus.

The Q-system is a binary system for transgene expression, originally developed for *Drosophila* and mammalian cells [24, 25], that has also been demonstrated in *Caenorhabditis elegans* [26], zebrafish [27] and malaria mosquitos [28]. The Q-system utilizes regulatory genes from the *Neurospora crassa* quinic acid gene cluster. The *N. crassa* quinic acid genes contain binding sites named QARE (QA response element) [29], referred to as QUAS when used in synthetic expression systems. Here, a synthetic expression system was developed for *P. chrysogenum*, by exploring components from the Q-system [30]. In this system, the synthetic TF (STF), consisting of the QF (qa-1F) DNA-binding domain (DBD) from the TF that regulates the quinic acid gene cluster of *N. crassa* which was fused to the *Herpes simplex* virus VP16 activation domain (AD) [31] and GFP with the SV40 nuclear localization signal (NLS) [32]. We demonstrated the function of this system by fluorescent reporters and showed that the production of penicillin could be controlled by introducing the QUAS sequences and the STF in the penicillin biosynthesis gene cluster. Taken together, our control device can serve as an excellent tool for studying and increasing fungal SM production and expressing of other genes of interest.

## Results and discussion

Engineering of production hosts requires robust and predictably performing gene expression tools. In this study, we set out to establish such tools for *P. chrysogenum* and to demonstrate their utility for the production of penicillin, implementing synthetic regulation for a SM cluster.

### Design of synthetic control devices

**Fig. 1:**
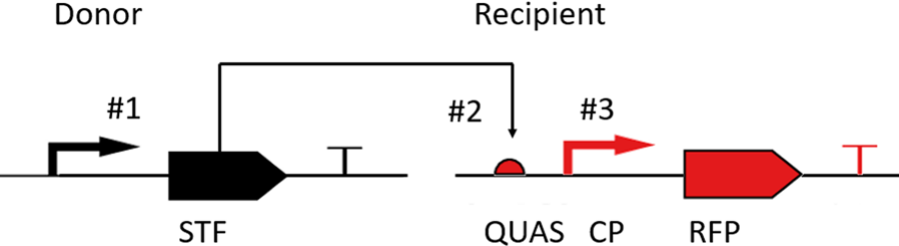
Schematic presentation of the control devices consisting of the donor (in black) and recipient (in red). The STF contained the QF DBD and the VP16 AD tagged with GFP-NLS, transcribed under the p*40S* or p*gndA* promoter (#1, black arrow). Control devices with 1, 5 and 11 QUAS elements (#2, red half circle) preceding various CPs (#3, red arrow) were evaluated. T’s present terminators of the expression cassettes. Elements varied are indicated by numbers and explained in Table 1

In order to design synthetic control devices for defined strength and expression profiles, components of the Q-system [30] were adapted for *P. chrysogenum*. The Q-system was chosen, as the DNA sequence to where the QF TF binds was relatively long (16 bp), which is needed in order to minimize pleiotropic effects and ensure a tight control. The control devices are defined as genetic systems where a STF controls the expression of a gene under a synthetic promoter containing a core promoter (CP) and binding sites for the synthetic transcription factor (STF). The strength of the control device is determined by: (1) the strength of expression of the STF; (2) the UAS element, which is the TF-specific binding site placed upstream of the CP; (3) the affinity of the DNA binding domain (DBD) of the TF to its UAS sequence; (4) the capacity of the activation domain (AD) to recruit the transcription machinery; and (5) the CP, which is necessary for assembly of the general transcription machinery and for initiation of transcription. In this work, the first three elements were investigated (Fig. 1).

The control devices consist of two transcriptional units, the donor for expression of the STF and the recipient with a reporter demonstrating the activity of the synthetic promoter (Fig. 1). This construct was integrated at the genomic site where the penicillin cluster normally is located in *P. chrysogenum*. In the strain used, DS68530, all the penicillin clusters had been removed [33]. In the control devices, the STF contained the DBD of the QF TF, fused to the VP16 AD and a GFP with an NLS (Fig. 1). Control devices with a modified QF (QF2 [25]) AD, appeared to possibly be toxic for *Penicillium*, as no correct transformants were obtained despite numerous trials. The GFP of the STF serves as an internal control which allows for corrections in growth and biomass differences. The STF controls the expression of the RFP reporter under a synthetic promoter containing QUAS elements upstream of a CP. CP strains (strains 4,5,7–11) express the donor with the STF but lack the QUAS elements upstream of the CP in the recipient. Six different CPs and three different QUAS elements were tested. In addition, two different promoters, p*40s* and p*gndA*, were used for expression of the STF. Strains characterized by measurement of fluorescence of control devices are listed in Table 1.

**Table 1:** Strains characterized by fluorescence measurements

No.	Description of strain	Number of QUAS elements (#2)	Promoter for expression of RFP (#3)	Gene associated with promoter for expression of RFP
1	40s_1xQ_pcbC	1	*Pc*_pcbC CP	Pc21g21380
2	40s_5xQ_pcbC	5	*Pc*_pcbC CP	Pc21g21380
3	40s_11xQ_pcbC	11	*Pc*_pcbC CP	Pc21g21380
4	40s_pcbC_CP	–	*Pc*_pcbC CP	Pc21g21380
5	gndA_pcbC_CP	–	*Pc*_pcbC CP	Pc21g21380
6	40s_5xQUAS	5	–	–
7	40s_pcbAB_CP	–	*Pc*_pcbAB CP	Pc21g21390
8	40s_penDE_CP	–	*Pc*_penDE CP	Pc21g21370
9	40s_phl_CP	–	*Pc*_phl CP	Pc22g14900
10	40s_nirA_CP	–	*An*_nirA CP	AN0098
11	40s_ura3_CP	–	*Sc*_ura3 CP	YEL021 W
12	40s_5xQ_pcbAB	5	*Pc*_pcbAB CP	Pc21g21390
13	40s_5xQ_penDE	5	*Pc*_penDE CP	Pc21g21370
14	40s_5xQ_phl	5	*Pc*_phl CP	Pc22g14900
15	40s_5xQ_nirA	5	*An*_nirA CP	AN0098
16	40s_5xQ_ura3	5	*Sc*_ura3 CP	YEL021 W
17	40s_5xQ_reverse_pcbC	5	*Pc*_pcbC CP	Pc21g21380
18	gndA_5xQ_pcbC	5	*Pc*_pcbC CP	Pc21g21380
19	gndA_5xQ_nirA	5	*An*_nirA CP	AN0098
20	gndA_5xQ_ura3	5	*Sc*_ura3 CP	YEL021 W
21	40s_pcbC full	–	*Pc*_pPcbC	Pc21g21380
22	40s_pcbAB full	–	*Pc*_pPcbAB	Pc21g21390

Elements varied are marked with numbers in Fig. 1. The STF transcribed under the p*40S* (An11g02040) promoter (#1) or the p*gndA* (AN0465) promoter (strains 5, 18–20). All strains were derived from DS68530

**Fig. 2:**
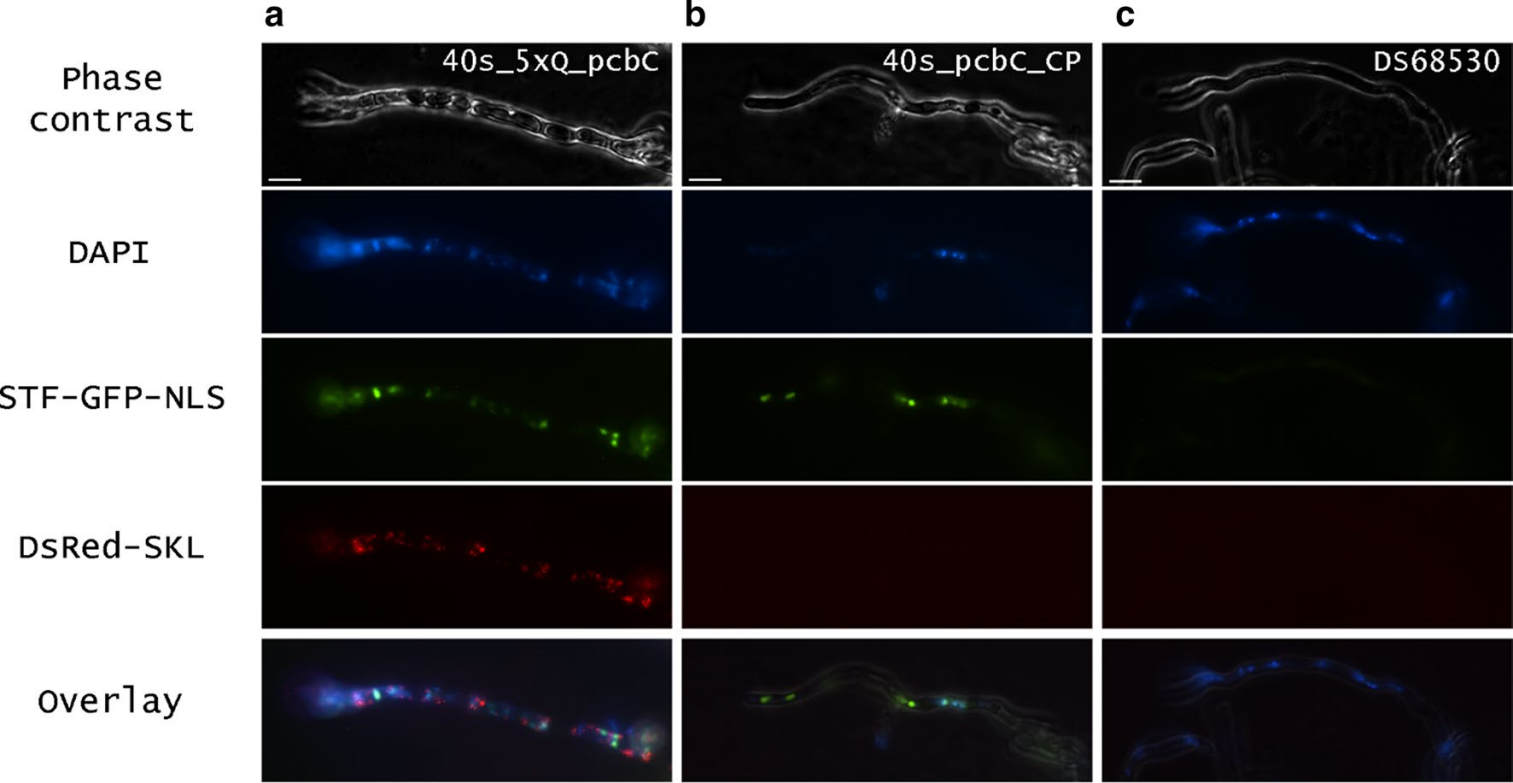
Fluorescence microscopy images of a filament of strain no. 2; 40s_5xQ_pcbC (**a**) expressing the STF (containing a GFP-NLS tag) under p*40s* and RFP under a synthetic promoter containing 5xQUAS upstream *Pc*_pcbc CP, strain no. 4; 40s_pcbC_CP (**b**) expressing the STF but no RFP as there are no QUAS binding sites upstream of the *Pc*_pcbC CP, and the parent strain DS68530 (wt, **c**) not expressing fluorescent proteins. Scale bars represent 10 µm

The control devices can be easily visualized due to the fluorescent protein reporters with different localization tags (Fig. 2). The STF containing GFP with an NLS tag was localized in the nucleus and the RFP with the SKL tag [34] localizes to peroxisomes of the cells. Upon fluorescence microscopy imaging of strains expressing the control devices but lacking the QUAS elements upstream of the CP, only GFP was seen (Fig. 2b), whereas strains with QUAS elements had green fluorescent nuclei and red fluorescent peroxisomes (Fig. 2a). The nuclear localization of GFP was confirmed by DAPI staining (Fig. 2c). The fluorescent imaging confirmed that all control device encoding genes were expressed and that the control device worked as designed.

**Fig. 3:**
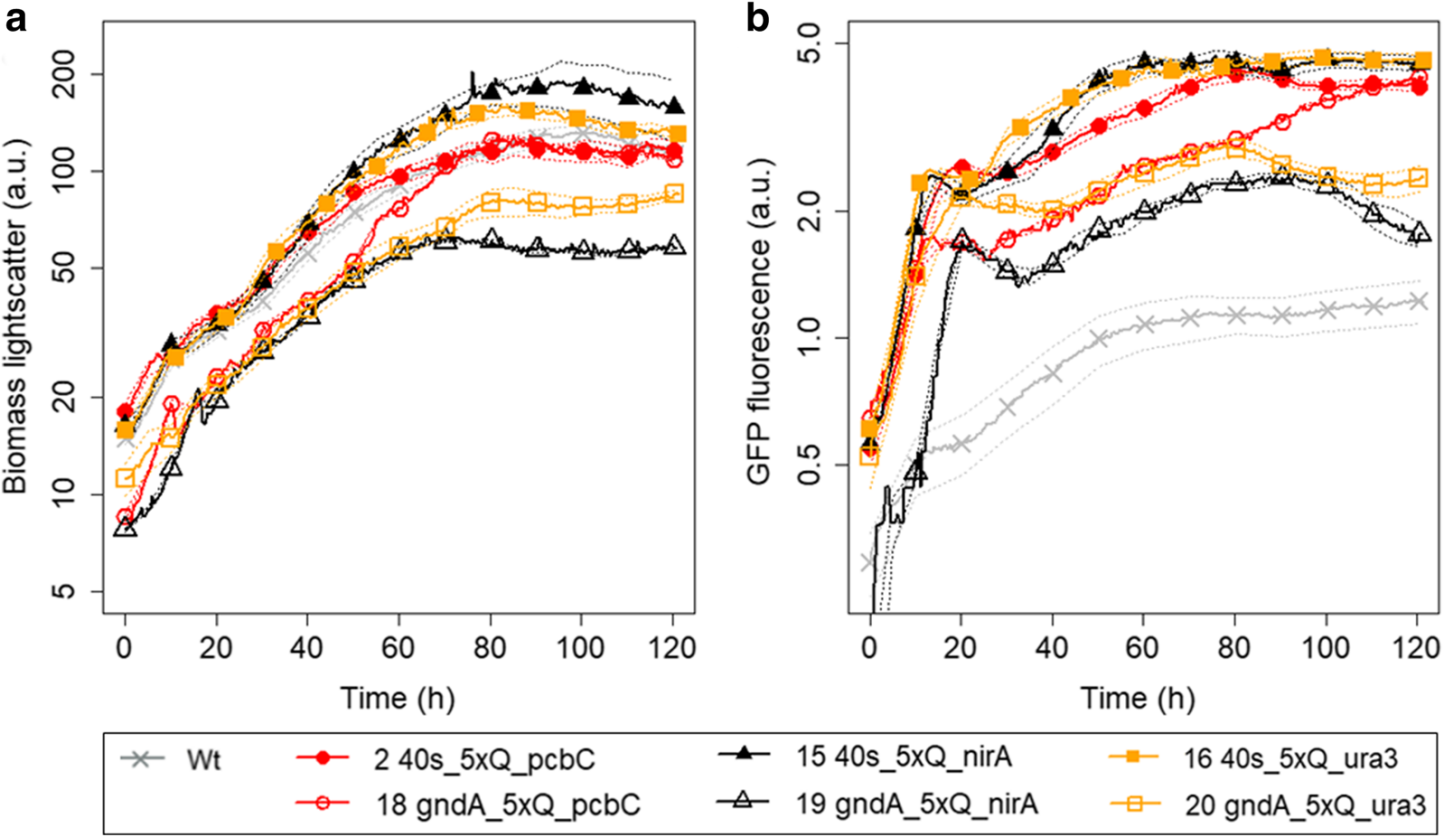
Development of biomass (**a**), GFP fluorescence (**b**), GFP fluorescence/biomass over time of selected *P. chrysogenum* strains containing different synthetic control devices, shown on logarithmic scales. Strain numbers and core promoters of strains are marked in the figure legend. The promoter used for expression of the STF was p*40S* or p*gndA* (marked in legend). Background fluorescence of parental strain (Wt) shown in grey (x plot symbols). Solid lines indicate mean values for at least 3 independent cultures, the dashed lines show the standard error. For data of all strains, see Additional file 1

The BioLector microbioreactor system with online monitoring of scattered light and fluorescence was used for assessing the performance of the control devices in *P. chrysogenum*. This system has previously been used to characterize bacterial [35] and yeast [36] fermentations as well as expression of fluorescent proteins under control of various promoters in *P. chrysogenum* [6]. Initially, we validated that no clear difference in exponential growth rate was seen among the strains evaluated and the wt strain (Fig. 3a and Additional file 1). The exponential growth rate determined during the first 60 h of cultivation was 0.031 ± 0.002 for all strains. An increase in biomass was observed during the first ~ 80 h of cultivation, after which the biomass remained constant or even decreased. None of the strains characterized in this study demonstrated any visible physiological changes during growth on liquid or solid medium. At the end of some of the cultivations, the mycelia clearly formed clumps, which likely explains the variability between some cultures seen after 80 h. It should be noted that the correlation between optical density and biomass concentration of filamentous fungi is linear only during the exponential growth phase (reviewed by Gibbs et al. [37]). Morphological changes after substrate depletion were for *Kluyveromyces lactis* cultures reported to influence biomass measurement [38], thus this is likely to also affect the late measurement of biomass for *P. chrysogenum* cultures.

The consumption of carbon sources of the growth medium (containing 5 g/l glucose and 36 g/l lactose) was measured after 24 and 120 h of cultivation for a few strains. After 24 h, all glucose was consumed, but around 12 g/l of lactose was still left in the medium after 120 h. The control device strains expressing fluorescent proteins were also tested in medium with glycerol or glycerol and lactose as carbon source. Glycerol is a non-fermentable carbon source that does not lead to glucose repression, while lactose is commonly used for production of SMs in fungi [3, 5, 6]. While growth on glycerol was challenged and no difference in expression of the control devices tested was seen in medium with glycerol and lactose, we did not pursue the testing of the production strains in different media. The growth of all the strains in the Biolector platform was reproducible and the biomass formation was not influenced by expression of any of the control devices tested.

### Tuning expression by varying the expression of the STF

**Fig. 4:**
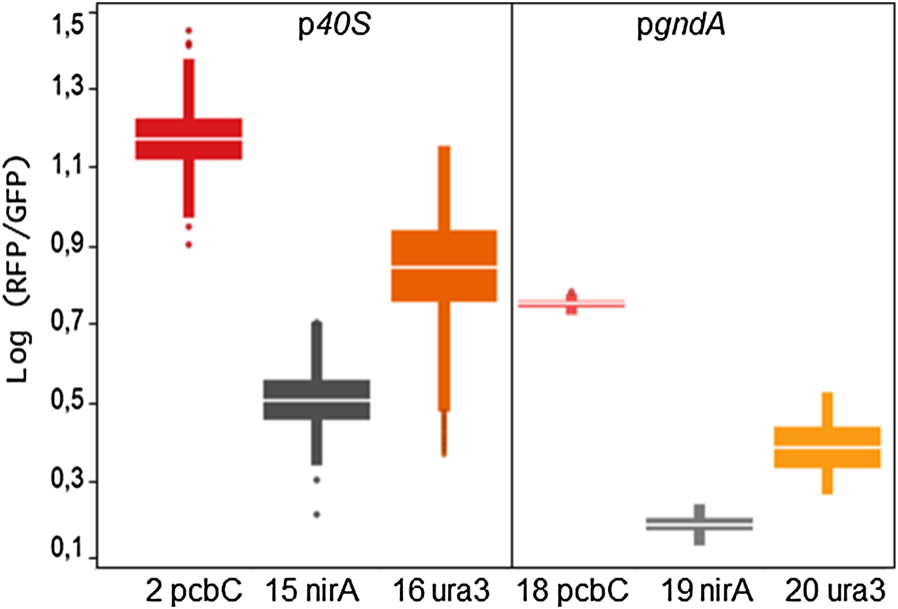
Ranking of the expression of 6 control devices. The activity is expressed as the logarithmic values of the averaged RFP/GFP fluorescence ratios measured during the time window of 40–80 h of growth in the BioLector system. All devices contain 5xQUAS; promoters for expression of the STF (on top) and CPs (on bottom) marked in figure. Box plot shows data of at least 3 independent cultures

Two different promoters were used to drive expression of the STF, the promoter of An11g02040 (p*gndA*) and of AN0465 (p*40*S, Fig. 3b, Table 1). Both promoters originate from *A. nidulans* and were previously validated in *P. chrysogenum* [6, 39]. The constructs with p*40S* for expression of the STF (strains 2, 15 and 16) gave 2–3 times higher expression of RFP/GFP, compared to the construct where p*gndA* was used (Fig. 4, strains 18–20) and were therefore chosen for further work. During the time interval of 40–80 h, the expression of GFP under p*40S* was approximately 1.5–2× higher than the expression under p*gndA*. This showed that the control device functions as an expression amplifier, in line with earlier observations in *S. cerevisiae* [19].

**Fig. 5:**
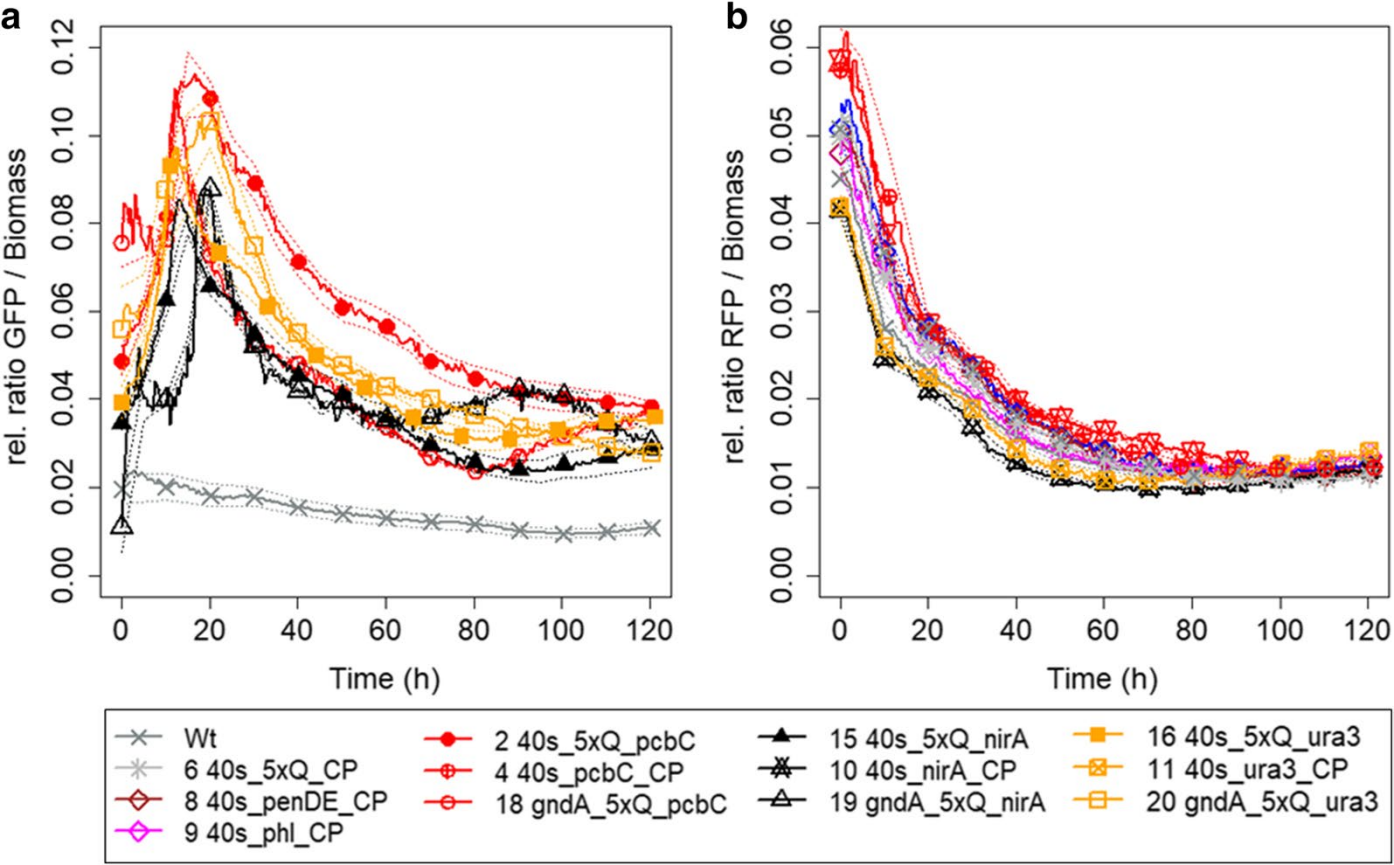
Relative ratio of GFP (**a**) or RFP (**b**) per biomass over time of selected *P. chrysogenum* strains containing different synthetic control devices. Strain numbers and core promoters of strains are marked in figure legend. The promoter used for expression of the STF was p*40S* or p*gndA* (marked in legend). Background fluorescence of parental strain (Wt) shown in grey (x plot symbols). Solid lines indicate mean values for at least 3 independent cultures, the dashed lines show the standard error. For data of all strains, see Additional file 1

The strength of the synthetic promoters (as measured by RFP fluorescence) was determined relative to the GFP expression of the strains to avoid variance caused by differences in growth. The expression of GFP was similar among the various strains with the same promoter for expression of the STF (Fig. 3b). The expression of GFP per biomass increased during the initial growth phase that lasted 10–20 h, depending on the strain and initial biomass concentration (Fig. 5a), after which the relative GFP expression decreased as more biomass was formed. The expression of GFP under p*gndA* was somewhat lower and showed a greater variability compared to the expression under p*40S* that was very similar among the different strains (Fig. 3b).

For the control device to function as anticipated, CPs should not be active by themselves, but the expression of RFP should be solely dependent on the expression of the STF. Furthermore, the QUAS element should not induce any expression by itself. The criteria set for the control devices were met: a strain with 5xQUAS elements upstream of the reporter showed no expression of RFP (Fig. 5b, strain 6; grey stars) and the strains lacking QUAS elements (strains 4 and 8–11) showed no expression of RFP (Fig. 5b).

### Tuning the strength of expression of the STF by varying the CP or number of QUAS elements

No CPs have previously been identified or validated in *P. chrysogenum*. Here, the 200 bps upstream region of the ATG of the penicillin cluster genes (Pc21g21370; *penDE*, Pc21g21380, *pcbC* and Pc21g21390; *pcbAB*) or Pc22g14900 (*phl)* was assessed as putative native CPs. *Phl* encodes a phenylacetyl-CoA ligase, involved in penicillin G and V production [40]. The CP sequences contain many putative CP elements (see Additional file 1) but no apparent similarities and they do not align. Nucleosome occupancy heatmaps of the recipient parts of the control devices drawn according to Kaplan et al. [41] were not found to correlate with the activity of the CPs (see Additional file 1).

**Fig. 6:**
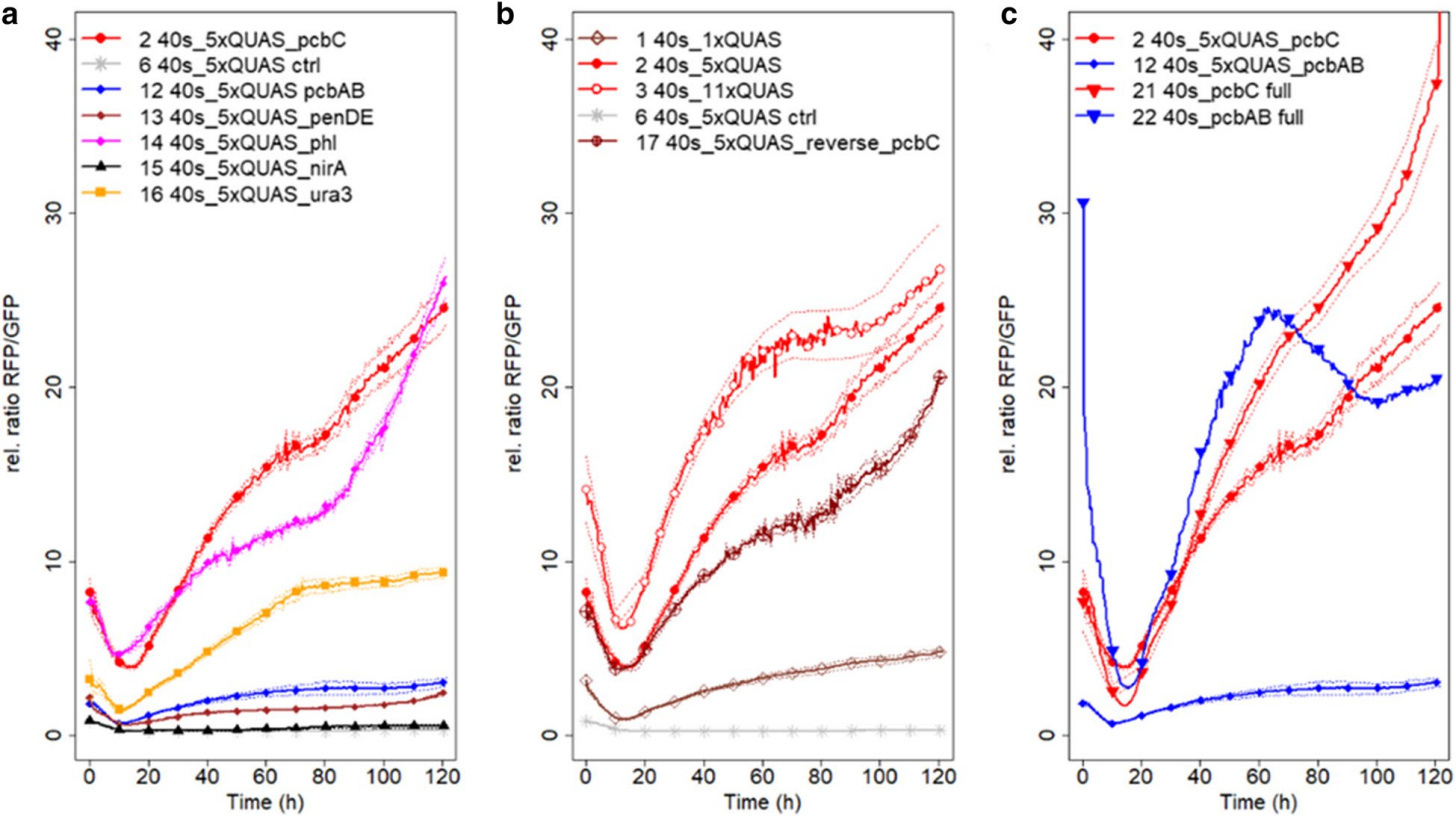
Development of RFP/GFP fluorescence over time during growth of selected *P. chrysogenum* strains. **a** The strains contain control devices with different CPs. **b** The strains express RFP under a synthetic promoter containing 1 (open diamonds), 5 (closed symbols, in 5′→3′ direction; open with a plus in 3′→5′ direction), 11 (open symbols) or no (stars) QUAS elements upstream of the *Pc*_pcbC CP. **c** The strains express RFP under the full promoters of *pcbC* (red triangles down) or *pcbAB* (blue triangles down) or under a synthetic promoter containing 5xQUAS elements upstream of the *Pc*_pcbC (red circles) or *Pc*_pcbAB (blue diamonds) CPs. Sold lines indicate mean values for at least 3 independent cultures, the dashed lines show the standard error. The difference in expression of RFP of all strains expressing functional control devices, was significant (p < 0.0001) compared to the base strain (strain no. 2, 40s_5Q_pcbC)

All CPs tested were shown to be functional as CPs in *P. chrysogenum* (Fig. 5a). By themselves (in strains 4, 5, 7–11, Fig. 5b) the CPs did not drive expression of RFP, but together with QUAS elements placed upstream, they formed functional synthetic promoters. In promoters with the QUAS element upstream the CPs, the constructs containing the CPs of *pcbC* and *phl* gave the highest expression, whereas the constructs with the CPs of the other penicillin cluster genes, *pcbAB* and *penDE* gave a maximal expression that was around 10× lower than the expression of the construct with the pcbC CP (Fig. 6a).

The expression of RFP under the control of 5xQUAS elements upstream of the *Sc*_ura3 CP was around twice as high as in a strain with the *An*_nirA CP downstream of the 5xQUAS element (Fig. 6a). This is in line with observations for *A. niger* strains, where these CPs were used in estradiol responsive constructs; the *Sc*_ura3 CP containing construct gave a much higher expression than constructs with the *An*_nirA CP [17]. The *nirA* gene encodes a transcriptional regulator mediating nitrate induction and is constitutively expressed at extremely low levels [42]. The *URA3* gene is commonly used as an auxotrophic marker in yeast and the *Sc*_*ura3* CP contains a TATA and a CCAAT box at around − 100 bps relative to the start codon (see Additional file 1). In *S. cerevisiae*, the full-length *URA3* promoter is reported to be relatively weak [43]. The *An*_nirA CP contains none of the known CP elements and was even suggested to be a repressor element, despite functioning as a weak CP [17]. Nonetheless, control devices with an expression ranging from very low to high is needed for balancing pathways. Here, we have shown that the expression of our devices can be varied by changing the CP unit of the control device.

### Benchmarking control devices against native promoters

Two of the control devices were benchmarked against native (full) promoters, the strong *pcbC* promoter, that is widely used for overexpression of genes in *P. chrysogenum,* and the less employed *pcbAB* promoter (Fig. 6c). The expression of the control device containing 5xQUAS upstream of the *Pc*_pcbC CP was similar to the expression under *pcbC* and *pcbAB* during the first 30 h, after which the expression was somewhat lower than the expression under the native promoters (Fig. 6c). In contrast, the expression of the control device containing 5xQUAS upstream of the *Pc*_pcbAB CP was at its peak (at 60 h) only about one-tenth of the expression of the native *pcbAB* promoter.

The expression of RFP under the *pcbC* or *pcbAB* promoter was similar during the first 65 h, after which the expression per GFP or biomass of the construct with *pcbAB* declined. Notably, the biomass of the strain containing the *pcbAB* promoter decreased at the end of the cultivation, while the biomass of the strain containing the *pcbC* promoter remained constant or decreased only later. The biomass measured in the BioLector microwells showed variability at the end of the cultivations, thus the difference between the strength of the *pcbAB* and *pcbC* promoter may not be significant under different conditions. In a previous study, it was observed that the *pcbAB* promoter was constantly much stronger than *pcbC* [6]. In this study by Polli et al. [6], 10 bps upstream of the start codon was lacking for both promoters, which may explain differences in expression.

The *pcbC* and *pcbAB* genes of *P. chrysogenum* face opposite directions and their intergenic region of around 1 kbps forms a bidirectional promoter. Both the *pcbAB* and *pcbC* genes are among the highest expressed [44] and the *pcbAB* and *pcbC* promoters were shown to be among the strongest tested to drive expression of a fluorescent protein [6]. In chemostat cultivation, the expression of *pcbAB and penDE* was reported to be approximately 80% or 40% of the expression of that of *pcbC*, respectively [44]. *Phl* is expressed at relatively low levels; the expression during glucose-limited chemostat cultivation was approximately 4% of that of *pcbC* in a high penicillin producing strain, containing 8 copies of the penicillin gene cluster [44]. Thus, there was no correlation between the reported native expression of the genes from which the CPs originate and the synthetic promoters containing the respective CPs. This is likely due to native regulation being disturbed in CPs. Still, the best performing control devices constructed showed a strength similar to the strongest promoters known for *Penicillium*.

### Tuning expression by varying the QUAS element

The possibility to tune the expression levels is perhaps the most important feature of a control device. Modulation of expression by varying the number of UAS elements in the CP has been shown previously in various systems [19, 45–47]. Therefore, constructs with one, five or eleven QUAS elements upstream of the *Pc*_pcbC CP were evaluated. As expected, the number of QUAS elements had a direct influence on the level of expression of RFP (Fig. 6b). The promoter containing five QUAS binding sequences led to a final expression that was approximately 5-fold higher than a promoter with a single QUAS binding sequence. The expression under the construct with 11xQUAS binding sites was during the first 60 h about 50% higher compared to the 5xQUAS construct (Fig. 6b), but leveled off after around 60 h, leading to a final expression similar to that of the construct with 5xQUAS. In line with these observations, several previous studies [19, 46, 47] report that the number of UAS elements influences the strength of expression, but the expression levels off or even decreases after a certain number of repeats. It may be that the availability of transcription factors becomes limiting or that the increased amount of RNA cannot be translated into protein due to lack of available amino acids or energy. The observation that the expression in the construct with 5xQUAS was about 5 times the expression of the construct with 1XQUAS suggests that the short linker (2 bp) between the binding sites was not limiting the binding of the STF.

The bidirectional promoter of the *qa*-*1F* and *qa*-*1S* genes of the quinic acid cluster of *N. crassa* contains a common QUAS element [29]. This study confirms that the QUAS elements function in both directions (Fig. 6b), which is highly applicable for construction of synthetic pathways. Bidirectional promoters are very common in SM clusters, but the expression of the bidirectional genes may vary [10, 44]. When the 5xQUAS containing element was placed in the reverse direction (3′→5′) upstream of the *Pc*_pcbC CP, the expression of RFP was identical during the first 25 h of growth, after which it was lower compared to the construct with the QUAS elements in the original direction. This amounted to about 80% of the expression of the construct with the QUAS elements in 5′→3′ direction during the time interval of 40–120 h. The strength of the different variants of the QUAS elements (see additional file 1; different repetitions of the GGRTAANNNNTTATCC sequence were designed to avoid spontaneous recombination), was not studied but may influence the overall strength of the control device and be more pronounced in one direction compared to the another. Quite some variability is seen in the QUAS elements of the native quinic acid pathway genes of *N. crassa*, leading to a large difference in affinity towards QF [29]. In line with this, Kiesenhofer et al. [48], showed that inverting repeats of *cis* elements in the *T. reesei cbh1* promoter can be used to modulate expression. As our device containing 5 slightly varying repeats of the QUAS element showed an expression of five times the construct containing only one element it may be assumed that the difference in affinity was not greatly influenced by the variability of the sequence and that the expression of the device can be tuned by altering the number of the binding sites.

### Regulation of the penicillin cluster using the control device

**Fig. 7:**
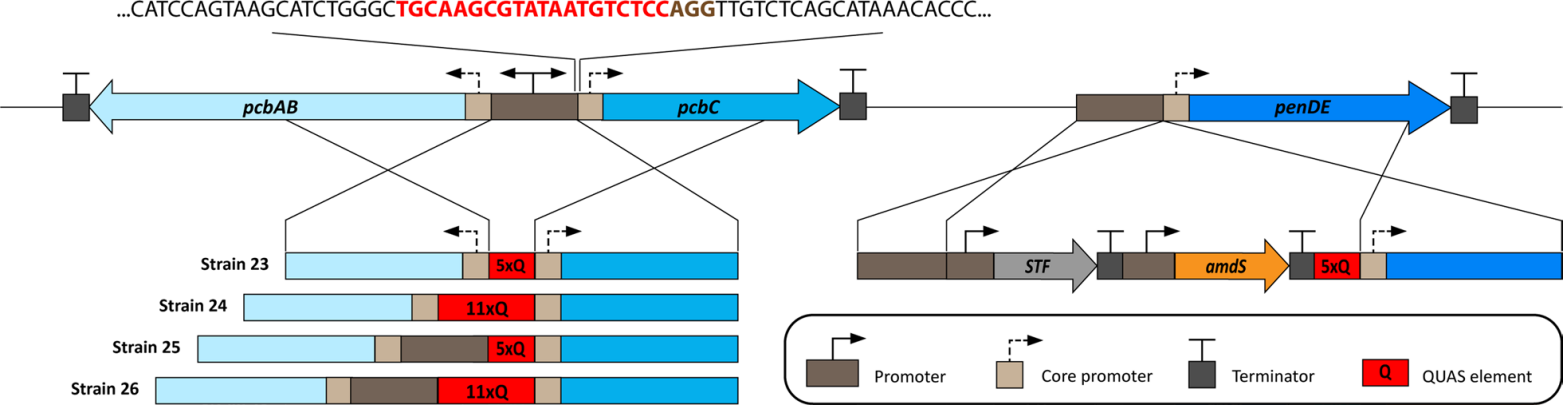
Schematic representation of CRISPR/Cas9 mediated co-transformation of the synthetic control device into the penicillin cluster. The marker-free donor DNA carried QUAS elements with flanking regions for homologous recombination at the *pcbAB*/*pcbC* locus. The *amdS* marker carrying integration cassette delivers the STF and a 5xQUAS element upstream of the core promoter of *penDE* gene

The penicillin cluster of DS54468 (1 copy) was placed under the regulation of the control device. Full-length promoters or CPs as well as different number of STF binding sequences (5× and 11× repeated) in constructs for expression of the penicillin synthesis genes were explored (Table 2, Fig. 7). All strains contained a STF driven by p*40S* and a construct where the 5xQUAS element is put upstream of the *Pc*_penDE CP that drives *penDE*. Penicillin V production under synthetic regulation was successful in all strain variants and the Penicillin V titers achieved were dependent on the constructs used for expression of *pcbAB* and *pcbC* (Table 2), reaching levels also observed with the native promoter. After 5 days, all cultures had reached a biomass of ~ 15 g/kg broth.

**Table 2:** Penicillin V production in shake flask cultures and characteristics of strains where the penicillin cluster was put under the control of the synthetic transcriptional factor

Strain no.	Promoter for expression of *pcbAB*	QUAS elements of *pcbAB*-*pcbC* locus	QUAS elements of *penDE* locus	Penicillin V titer (g/L)^a^		
				After 3 days	After 5 days	After 7 days
DS54468	*Pc*_pcbAB	–	–	0.34 ± 0.016	0.48 ± 0.023	0.48 ± 0.006
23	*Pc*_pcbAB CP	5xQUAS	5xQUAS	0.04 ± 0.001	0.06 ± 0.000	0.07 ± 0.002
24	*Pc*_pcbAB CP	11xQUAS	5xQUAS	0.05 ± 0.002	0.08 ± 0.001	0.14 ± 0.005
25	*Pc*_pcbAB full	5xQUAS	5xQUAS	0.14 ± 0.002	0.26 ± 0.002	0.33 ± 0.011
26	*Pc*_pcbAB full	11xQUAS	5xQUAS	0.20 ± 0.003	0.38 ± 0.003	0.49 ± 0.006

All strains were derived from DS54468

^a^Mean ± SEM of 3 biological replicates, with 3 technical replicates each

The characterization of the control devices in microbioreactors, proved to give excellent indications for how the devices function in the production strains and conditions. In strains where only the respective CPs drive *pcbAB* and *pcbC* (strains 23 and 24), less Penicillin V was produced compared to the parental strain (DS54468), likely due to the *Pc_*pcbAB CP being a very weak CP (Fig. 6a). The *Pc_*penDE CP was also shown to be rather poor, only about 13% compared to the device containing *Pc_*pcbC CP (Fig. 5a). However, the transcription of *penDE* in native penicillin production strains is also much lower than expression of *pcbAB* or *pcbC* [44].

During the whole experiment, 11xQUAS carrying constructs overperformed their 5xQUAS counterparts both in *Pc*_pcbAB CP (strain 23, 24) and *Pc*_pcbAB full promoter (strain 25, 26) utilizing setups. In strain 25 with a full promoter driving *pcbAB* and the synthetic control devices with 5xQUAS and respective CPs upstream of *pcbC* and *penDE*, the production was ~ 70% of that of the parental strain (DS54468). The final Penicillin V titer of strain 26 with 11xQUAS was ~ 150% of the titer produced with strain 25 containing 5xQUAS and reached the production levels of the parental strain (DS54468). The use of constructs with a high number of QUASs combined with CPs that would allow a higher and faster expression of all the penicillin cluster genes may be expected to lead to strains with increased penicillin production rate. The scalability demonstrated by fluorescence reporters was verified by synthetically controlling the production of penicillin.

## Conclusions

Filamentous fungi are attracting increasing interest as biotechnological production hosts, but efficient genetic tools for exploitation were lacking. Therefore, we successfully developed a modular, synthetic control device for *P. chrysogenum* and demonstrated its function through regulation of the penicillin cluster. The strength of the control device was altered by altering the expression of the synthetic transcription factor (STF), the core promoter downstream the QF Upstream activating sequence (QUAS), or the amount of QUAS elements, leading to an expression ranging from hardly detectable to an expression similar to that of highest expressed native genes. A gene cluster may demand an expression tuned individually for each gene, which is a great advantage provided by this system. We anticipate that these well-characterized and robustly performing control devices can be highly useful tools in the development of filamentous fungi as production hosts.

## Methods

### Fungal strains and culture conditions

*Penicillium chrysogenum* DS68530 (∆Penicillin-cluster, ∆hdfA, derived from DS17690) [33, 49] and DS54468 (1× Penicillin-cluster, ∆hdfB, derived from DS47274) [50] strains were kindly provided by Centrient Pharmaceuticals B.V., former DSM Sinochem Pharmaceuticals, the Netherlands.

Fungal strains were purified and grown on solid complex or transformant selection medium with 0.1% acetamide as a sole nitrogen source [6, 51]. Mycelium from the complex medium was collected for long term storage of strains on rice grains or for microscopy analysis. Spores (immobilized on 25 rice grains) or biomass grown on solid complex medium for 2–3 days until sporulation, were used to inoculate 25 or 10 ml (using spores or biomass, respectively) SM production medium [6]. Cultures were incubated for 42–50 h in a rotary incubator at 200 rpm at 25 °C.

For BioLector analysis and analysis of growth in FlowerPlate (MTP-48-B) wells, this pre-grown mycelium was diluted 8 times in fresh SM production medium. The 1 ml cultures were grown in the BioLector microbioreactor system (M2Plabs, Germany), shaking at 800 rpm at 25 °C. In the BioLector, biomass was measured via scattered light at 620 nm excitation without an emission filter. The fluorescence of GFP-NLS and DsRed-SKL (for simplicity referred to as GFP and RFP in the text) was measured every 30 min with 486/589 nm excitation filter and 510/610 nm emission filter, respectively. In contrast to our previous work [6], the wavelength used for measuring RFP fluorescence was optimized for DsRed. For measurements of carbon consumption, the experiment was disrupted after 24 h and contest of wells was analyzed by HPLC (Shimadzu Prominence, Japan) using an HPX-87H column (Shimadzu, Japan), 0.005 M H_2_SO_4_ with a flow rate of 0.6 ml/min on 65 °C. All experiments were conducted in at least technical 4 replicates, of at least 2 different biological replicates. The data obtained from the BioLector experiments were analyzed using the TIBCO Spotfire Software (TIBCO Software Inc., USA) and presented using RStudio and the Plotrix package.

For penicillin fermentation, strains with STF and the Q-system regulatory elements integrated in the penicillin cluster were grown in YGG medium for 24 h, after which the cultures were diluted 8 times into penicillin production medium supplemented with 2.5 g/L phenoxyacetic acid, mediums prepared as described previously [52]. Supernatant samples for HPLC analysis were taken after 3, 5 and 7 days and extracellular Penicillin V titers were determined by UHPLC (Shimadzu Nexera UHPLC, Japan) using a Shim-pack XR-ODS 2.2 (75 mm L × 3 mm i.d.) column operating at 40 °C according to Weber et al. [52].

### Construction of expression cassettes for control devices

PCR amplifications were conducted using KAPA HiFi HotStart ReadyMix (Roche Diagnostic, CH) or Phusion High-Fidelity DNA Master Mix (Thermo Fisher Scientific, USA), for primers see Additional file 1. The MoClo modular cloning system [53] was employed for construction of all expression cassettes (Figs. 1, 6a; Tables 1, 2 for more details see Additional file 1). Flanking regions of approximately 800 bps were designed for integration of the expression cassettes at the locus of the deleted penicillin cluster of DS68530 by in vivo homologous recombination. Internal BsaI, BpiI and in most cases also DraIII recognition sites of the DNA elements were removed during the cloning. A modified protocol using the FastDigest versions (Thermo Fisher Scientific, USA) of the BsaI and BpiI restriction enzymes were used with an initial 10 min digestion, 20–50 cycles of digestion and ligation (37 °C for 2 min, 16 °C for 5 min), followed by a final digestion step and a heat inactivation step, was used for most assemblies, instead of the standard MoClo protocol.

The endogenous elements for the expression cassettes constructed in this study, were amplified by PCR from genomic DNA of *P. chrysogenum* DS54468. The *amdS* selection cassette used was described previously [54]. The 138 bps Sc_*ura3* CP amplified from genomic DNA of *S. cerevisiae* CEN. PK, is slightly longer compared to the version used by Pachlinger et al. [17]. The DsRed-SKL gene was amplified from the pJAK109 plasmid [54] while the promoter of *A. nidulans* ribosomal protein S8 (AN0465.2, referred to as *40S*) was amplified from pDSM-JAK108 [39]. The *gndA* promoter (Sequence ID: AM270223.1 32820 to 32040) from *A. niger CBS 513.88* was ordered as a synthetic DNA from IDT. The *GFP* was amplified from the pSpCas9-2A-GFP plasmid, kindly provided by Feng Zhang via Addgene (Plasmid #48138 [55]). The pAC-7-QFBDAD plasmid, used for amplification of the QF DBD was kindly provided by Christopher Potter, via Addgene (Plasmid #46096 [25]). The plasmid pVG2.2 used as a template for the VP16 AD was a gift from Vera Meyer [12]. The 94 bps long *An_*NirA CP (identical to the sequence used by Pachlinger et al. [17]) as well as the cassettes containing 1 or 5xQUAS sequences, were ordered as oligos that were annealed before assembly to level 0 vectors, the initial building blocks used in the MoClo system. The repetitions of the QUAS elements were designed to contain some variability as the genetic stability of *P. chrysogenum* strains was an initial concern. The 11xQUAS carrying plasmid was constructed with the assembly of three units of annealed oligos (see Additional file 1). The design was for creating a 15xQUAS containing part, but this was proven to be difficult for *E. coli* to assemble, as some QUAS sequences were looped out during the construction. The sequence of the actual 11xQUAS part constructed can be found in Additional file 1.

### Construction of a Q-system controlled penicillin production strain

The 5 or 11xQUAS elements were inserted in the intergenic region between the *pcbAB* and *pcbC* genes (leaving 200 bp CPs upstream each gene) of the penicillin cluster of DS54468 (Fig. 6, strains 23, 24) using co-transformation and the CRISPR/Cas9 technology described previously [54, 56]. Strains where the QUAS elements were inserted 200 bps upstream the *pcbC* gene but leaving the *pcbAB* promoter intact were created in a similar manner (Fig. 7, strains 25, 26). The integration of the marker-free dDNA was facilitated with in vitro preassembled CRISPR-Cas9 ribonucleoproteins where the sgRNA was targeting the TGCAAGCGTATAATGTCTCC**AGG** sequence at the boundary between the promoter of *pcbAB* and the CP of *pcbC*.

The dDNA for integrating the 5xQUAS elements upstream of the CP of *penDE* also contained the STF and an *amdS* marker (YN2_71, Additional file 1: Table S4). One μg plasmid containing ~ 1 kbp homologous 3′ and 5′ flanking regions for integration upstream to *penDE* was digested with DraIII before co-transformation with marker-free DNA. 5 μg marker-free dDNA cassette carrying plasmid (YN1_81, YN1_82, YN1_77, YN1_80 for strains 23, 24, 25 and 26 respectively, see Table S4) were digested with KspAI and PaeI leaving ~ 1–2.5 kbp homologous flanking regions around the QUAS elements for creating *Pc*_pcbAB CP or *Pc*_pcbAB full promoter strains (Fig. 7). All dDNA cassettes were build using the MoClo system [53]. Correct clones were selected using colony PCR and confirmed by sequencing. The strains were purified through 3 rounds of sporulation before liquid culture cultivation.

### Copy number determination by qPCR analysis

Copy numbers of genes and constructs were determined using the MiniOpticon™ system (Bio-Rad, USA) for analyzing gDNA isolated as described before [49]. SensiMix™ SYBR mix HI-ROX (Bioline, UK) was used as a master mix for qPCR with 0.4 µM primers and 10 ng gDNA in a 25 µL reaction volume. Data were analyzed using the BioRad CFX manager software in which the C(t) values were determined automatically by regression [49]. Copy numbers were calculated from duplicate experiments with three technical replicates, using the γ-actin gene (Pc20g11630) as a control for normalization [49]. The efficiency of the primers used for the copy number determination was assessed through the use of four dilutions of gDNA. Primers used for *pcbC*, *penDE*, and *STF* copy number identification (see Additional file 1) on strains 23,24,25,26 are listed in Additional file 1. The γ-actin, *pcbC*, *PenDE* and STF showed efficiencies of 100.17% (R^2^ = 1.000), 102.86% (R^2^ = 0.993), 96.38% (R^2^ = 0.999) and 97.87% (R^2^ = 0.998), respectively. *P. chrysogenum* DS54468 and DS68530 strains were used as controls containing zero copies of STF and 1 or 0 copies of the penicillin gene cluster, respectively.

### Fungal transformations and analysis of transformants

Transformations of *P. chrysogenum* were performed as described previously [51], using about 1.5 µg of digested plasmid(s) for each transformation. The expression cassettes were digested with MreI or DraIII, that cut twice in the backbone of the MoClo vectors. For some transformations, the protoplasts were cryopreserved, based on the method described for *U. maydis* [57]. After the final washing step of the protoplast formation, the protoplasts were suspended in STC medium and diluted twice in cryopreservation medium; 20% PVP-40 (Polyvinylpyrrolidone 40 (C_6_H_9_NO)_40_) in STC buffer (1.2 M sorbitol, 50 mM CaCl_2_, 10 mM Tris-HCl pH 7.5).

To confirm the integration of the cassettes at the correct locus, colony PCR was performed using the Phire Plant Direct PCR Kit (Thermo Fisher Scientific, USA) or with standard PCR reactions using DNA extracted from the cells using Lysing Enzymes from *T. harzianum* (Sigma-Aldrich, UK). In addition, PCR products of selected transformants were sequenced. All strains that were analyzed by sequencing of the QUAS and the CP region contained all designed QUAS repetitions, thus no strain instability due to repetitive elements was observed. For some constructs, we however observed ectopic recombination, regardless of (the strains being of) *∆hdfA* background; these transformants were dismissed from the core study.

### Fluorescence microscopy

Transformants were examined using fluorescence microscopy after 4 days of growth on acetamide solid medium. A small amount of hyphae was taken from the peripheral zone of the colonies and suspended in phosphate-buffered saline (58 mM Na_2_HPO_4_; 17 mM NaH_2_PO_4_; 68 mM NaCl, pH 7.3). Samples analyzed for nuclear localization were stained with 4′-6-diamidino-2-phenylindole (DAPI) (Sigma-Aldrich, UK) at 1 μg/ml in PBS buffer for 20 min. Samples were examined with Nikon Ti-E microscope (Nikon Instruments, Tokyo, Japan) equipped with Hamamatsu Orca Flash 4.0 camera with 100× objective, numerical aperture: 1.45. refractive index: 1.515. Pictures were taken using phase contrast, DAPI, FITC (GFP) and TRITC (RFP) filters. Strains no. 2, 4, 5, 10, 11, 15–20 were examined with fluorescence microscopy.

## Supplementary information

**Additional file 1.** Additional tables and figures.

## Data Availability

All data generated or analyzed during this study are included in this published article and its additional files.

## Abbreviations

SM: secondary metabolites
TF: transcription factor
DBD: DNA-binding domain
AD: activation domain
UAS: upstream activating sequence
STF: synthetic transcription factor
QUAS: QF Upstream activating sequence
