# Supplementary material for: Synthetic control devices for gene regulation in Penicillium chrysogenum
Source: Microb Cell Fact. 2019 Nov 18;18:203. doi: 10.1186/s12934-019-1253-3 (PMC6859608; doi:10.1186/s12934-019-1253-3)
Supplement: Supplementary file 1 — Additional file 1. Additional tables and figures. [file 12934_2019_1253_MOESM1_ESM.docx]

Additional file 1. Additional tables and figures.

Synthetic control devices for gene regulation in *Penicillium chrysogenum*

László Mózsik, Zsófia Büttel, Roel A.L. Bovenberg, Arnold J.M. Driessen, Yvonne Nygård

Figure S1: Biomass formation (scattered light) and fluorescence measured from all strains and medium (background fluorescence)

Figure S2: Nucleosome occupancy profile heatmap of the synthetic promoters

Figure S3: Strain verification through qPCR

Table S1: Characteristics of core promoters (CPs) used in the study

Table S2: Sequences of synthetic units used for cloning

Table S3: Primers used for amplification of PCR products used for cloning

Table S4: MoClo plasmid construction

Table S5: Primers used for qPCR


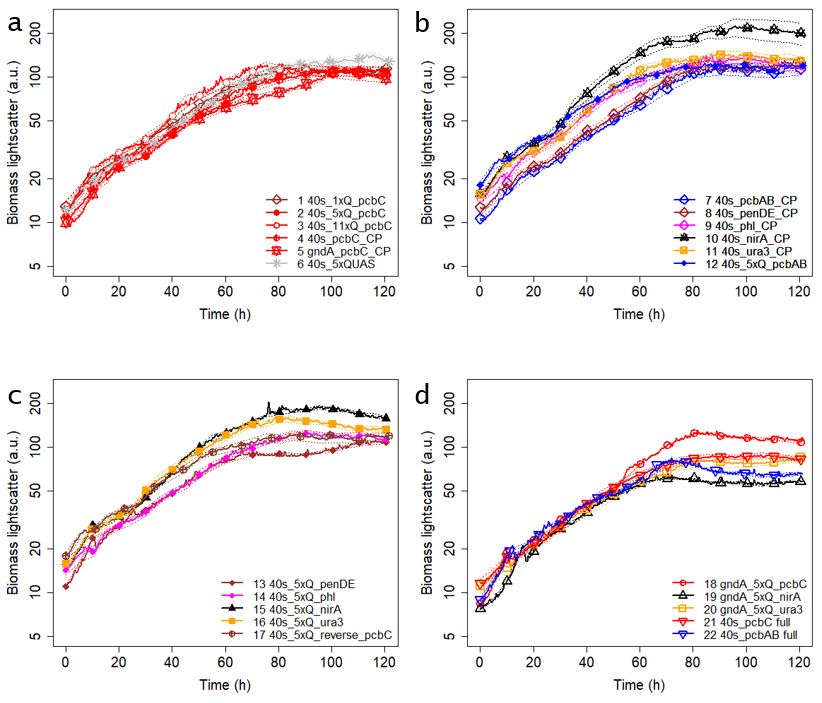

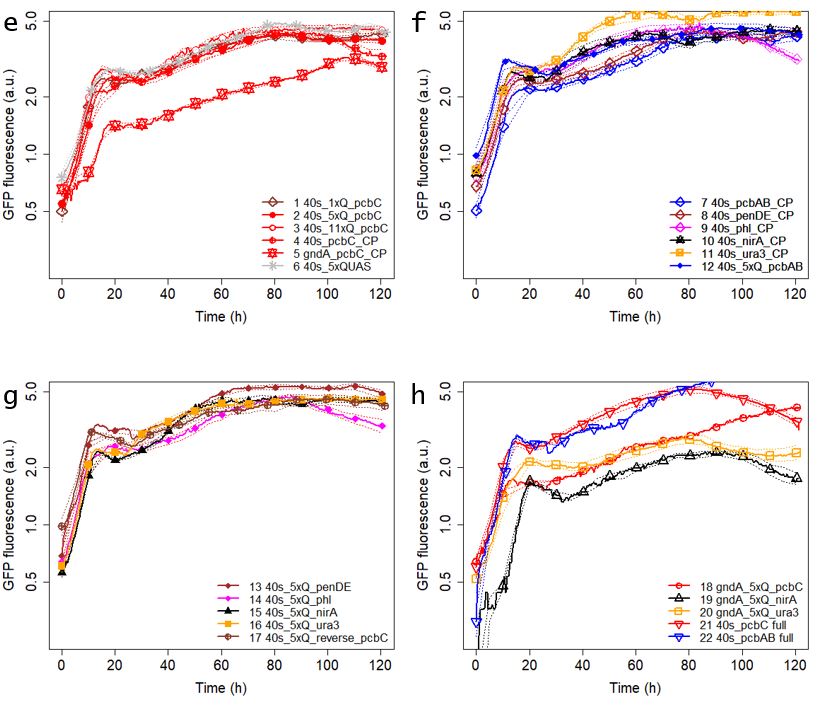


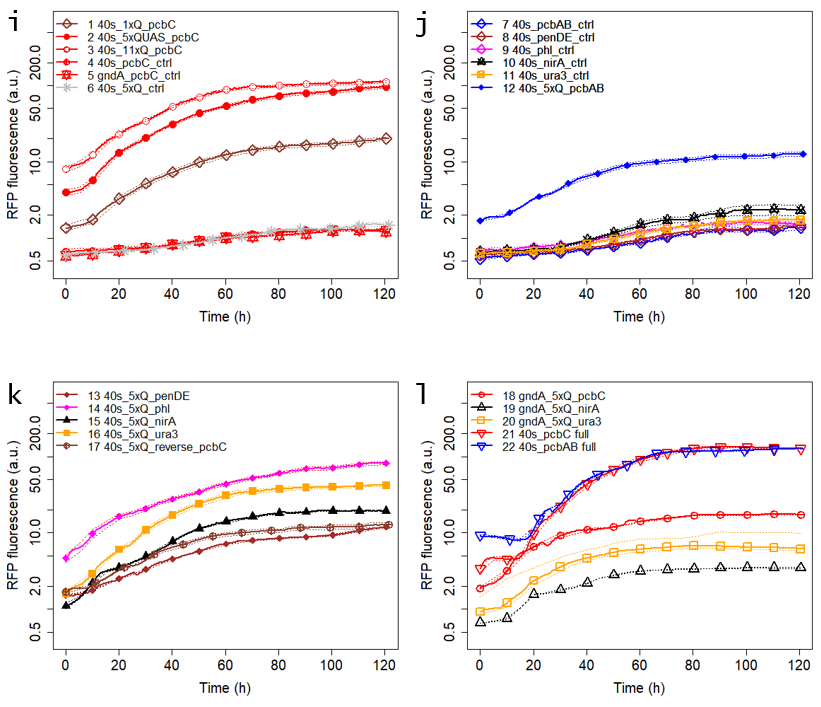


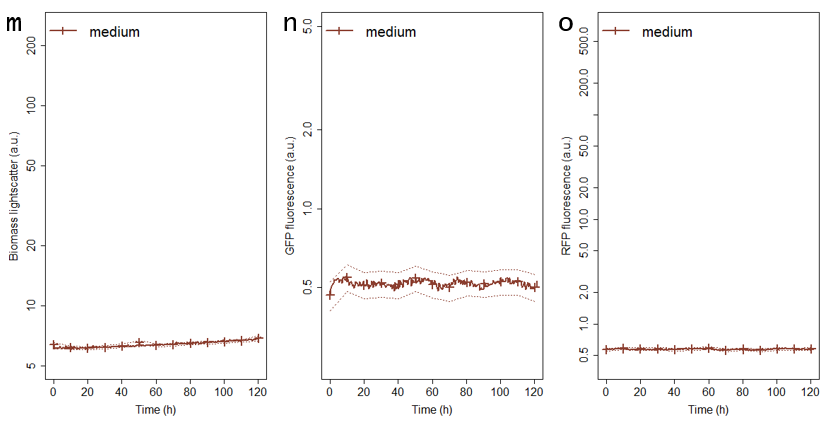


Figure S1: Development of biomass (a-d), GFP fluorescence (e-h), GFP and RFP fluorescence (i-l) over time of all strains, described in Table 1, , shown on logarithmic scales. Strain numbers and core promoters of strains are marked in figure legends. The promoter used for expression of the STF was p*40S* or p*gndA* (marked in legend). Background light scatter and fluorescence of medium shown in figure m-o, using same scales as in the corresponding figures showing data of strains (a-l). Solid lines indicate mean values for at least 3 independent cultures, the dashed lines show the standard error.


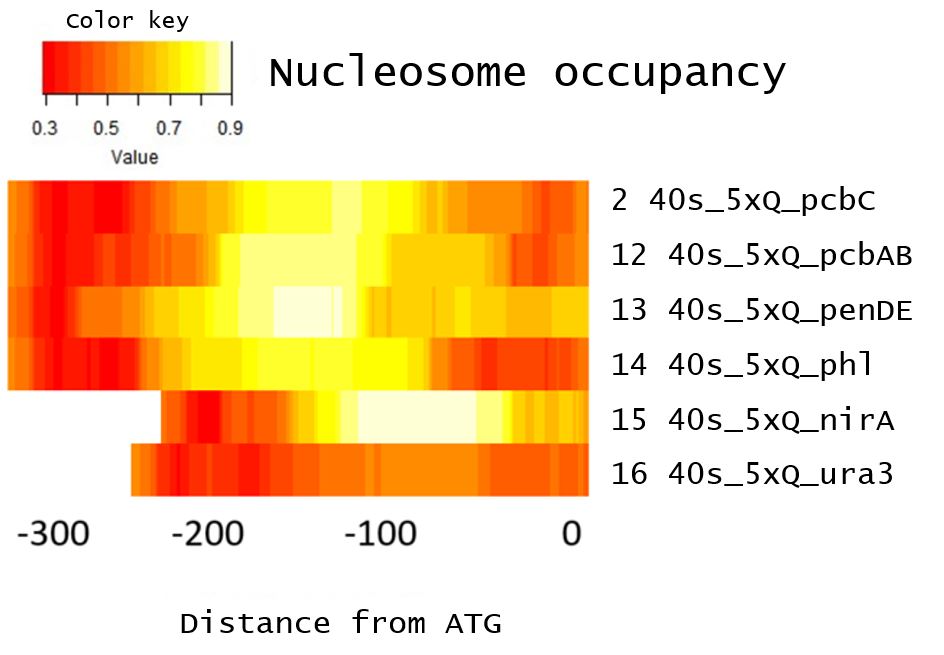


Figure S2. Nucleosome occupancy profile heatmap of the synthetic promoters, consisting of the CPs fused to 5xQUAS. The nucleosome occupancy was calculated according to Kaplan et al [1]. The lower nuclear occupancy of the *Sc*_ura3 CP may explain the relatively high expression of the synthetic promoter containing this CP (Figure 5a), despite an apparent difference in this CP in terms of GC content (Table S1). AT rich sequences have been reported to be associated with low nucleosome affinity and high promoter activity [2].


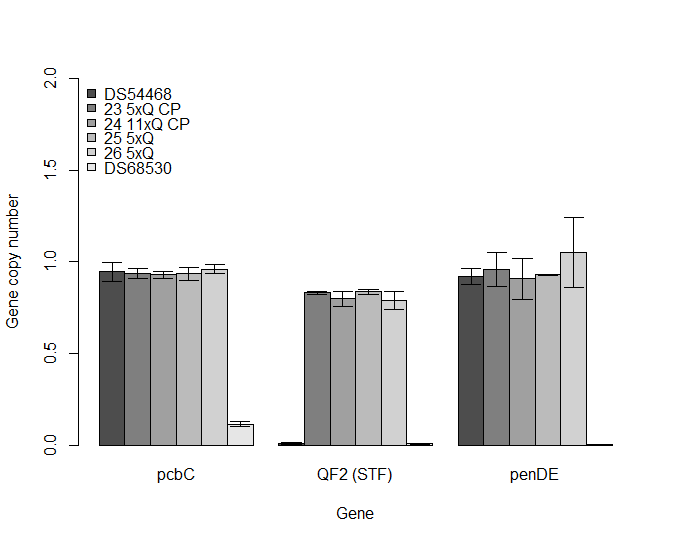


Figure S3: Strain verification through qPCR. Quantification of the copy number of the integrated donor DNA and two penicillin cluster genes (*pcbC* and *penDE*) in *P. chrysogenum* strains. Strains DS54468 (1xPen-cluster) and DS68530 (∆Pen, no penicillin cluster) were used as controls.

Table S1. Characteristics of core promoters (CPs) used in the study. Numbers indicate position of the CP element in relation to the start of the gene (ATG).

| **CP** | **Size** | **GC cont.** | **TSS** | **TATA** | **CCAAT** | **INR** |
| --- | --- | --- | --- | --- | --- | --- |
| *Sc*_ura3 | 94 | 40% | Multiple, -119 − -60 | -92, -90 | -98 | -99, -101 |
| *An*_nirA | 138 | 54% | Unknown | - | - | - |
| *Pc*_pcbC | 200 | 51% | -15 | -194 | - | -11, -107 |
| *Pc*_pcbAB | 200 | 50% | -106 | -94,-131 | -12, -18, -111, -149 | -94, -131, -164 |
| *Pc*_penDE | 200 | 55% | -73 | -104 | -107, -121 | 104, -122, -194 |
| *Pc*_phl | 200 | 50% | Unknown | -21 | - | -4, -19, -48, -122 |

**TSS = Transcription starting site, according to Šmidák et al. [3] or Losson et al. [4]. INR element = Initiator element; YYANWYY**

Table S2: Sequence of synthetic fragments used

| **ID** | **Description** | **Sequence (5' -> 3')** |
| --- | --- | --- |
| A | p*gndA* | TTGAAGACATGGAGTCTTGCGTTACGGGCGTATTTTGCTGCGGCCGGTGGTGCCCCTCCATGCCCCGCCATCTTTCAAAGCTCCTGGCGACGCCGTCATCTCCGAACATTCTCCCCCCAAAGGAATCAATTGGCAATTGGAGTCTAGTAAAGTGGTGTTTGTCATCAGTAAGGAGTTGGTGAAACTACAATCTTCCATCATGAAGAGAAGGGATATTTTTGGGGTTGTATTTTACGATGAAGGTACTGGAAATGGTGGGGGTTTTTATAGCAGTAGACAGTCAGTCAGTAAGTAGTATGCTTGTTGTATTACCCAAACCAGATCAATCCAAAGAAAGCCTGACAGACAGCCATCAATAGATACTACTTCGTACTATAGTTACCCACCTAACCATATTACTCAAAAAGCATCTATCTATCCGCGGGCTTCCATGCATGTCCCGGTAGCAAACTCCTCCCACCGGTGTAGTACTCTTTGGTTAGTAGTCTTGTTCACCGGAGGACTCTGCTCCTCTCCTGCTCAGGTGCTGCCCCGCCCTCCGTCCCACCATGACGGAAGAGATGCTCCGTAAGCCGTCCAGTTGCAACGAATCCTGCTCTGACATCTTCGAACGCCTTCTCCCTTTCGCTCGCTTCTCTGCCTCTTTCCTCTCTTCCCTTTCCTTCCCCTCCAAACTAAACCTTCCTCCTTTTCTCCATCATCCTCTAGGCAGTTGGTTCTTCCTGACTGTACATATATCCACCACCTCCCCCCTCTATTCTTCCACCTCTTCCATATCTCCTTCTCCAGAGTTCATACCCCCCACAATGATGTCTTCTT |
| B | 1xQUAS | TTGAAGACAAGGAGGGGTAATCGCTTATCCCCATAAGTCTTCTT |
| C | 5xQUAS | TTGAAGACAAGGAGGGGTAATCGATTATCCTCGGATAAACACTTATCCCAGGGTAATCACTTATCCCTGGATAAACAATTATCCTCGGGTAATCGCTTATCCCCATAAGTCTTCTT |
| D | 15xQUAS A | GTGAAGACTAGGAGGGGTAATCGATTATCCTCGGATAAACACTTATCCCAGGGTAATCACTTATCCCTGGATAAACAATTATCCTCGGGTAATCGCTTATCCTACGTTGTCTTCAC |
| E | 15xQUAS B | GTGAAGACAATACGGGGTAATCGATTATCCTCGGATAAACACTTATCCCAGGGTAATCACTTATCCCTGGATAAACAATTATCCTCGGGTAATCGCTTATCCTTGCTTGTCTTCAC |
| F | 15xQUAS A | GTGAAGACAATTGCGGGTAATCGATTATCCTCGGATAAACACTTATCCCAGGGTAATCACTTATCCCTGGATAAACAATTATCCTCGGGTAATCGCTTATCCCCATTTGTCTTCAC |
| G* | 11xQUAS | TTGAAGACAAGGAGGGGTAATCGATTATCCTCGGATAAACACTTATCCCAGGGTAATCACTTATCCCTGGATAAACAATTATCCTCGGGTAATCGCTTATCCTACGGGGTAATCGCTTATCCTTGCGGGTAATCGCTTATCCTCGGATAAACAATTATCCCAGGGTAATCACTTATCCCTGGATAAACAATTATCCTCGGGTAATCGCTTATCCCCATAAGTCTTCTT |
| H | *nirA* CP | TGAAGACAACCATCAGGGAAACACGCCGAGCGTCCTCCTCCGATAAGCATGCGCTGTCTTGGTCTGTCGCTGTCACCGAACATATCTGTCTGGGCTTGATTTACCATAATGAAGTCTTCT |
| I | *nirA* CP ctrl | TGAAGACAAGGAGCAGGGAAACACGCCGAGCGTCCTCCTCCGATAAGCATGCGCTGTCTTGGTCTGTCGCTGTCACCGAACATATCTGTCTGGGCTTGATTTACCATAATGAAGTCTTCT |

***=verified by sequencing**

Table S3: List of primers used for amplification of PCR products for cloning

| **ID** | **Description** | **Primer sequences (5'->3')** |
| --- | --- | --- |
| 1 | Pen 5’flank A | F:ACAGCGGAAGACAAGGAGCCTGCAGGATGGGCCTCCACAACCCTGCC R:TGAAGACAACGAATCTTCTATTCAATCTGATAAC |
| 2 | Pen 5’flank B | F:TGAAGACAATTCGGTGATGCAGCAAATAGCGA R:TGAAGACAATGAGGACAGGATATCACGCGTTACC |
| 3 | Pen 5’flank C | F:TGAAGACACCTCAGTCTTAAGACTTCTCACCTA R:ACAGCGGAAGACAAAGCGCTACCGTTTGTACCATCTGA |
| 4 | p*40S* A | F:TTGAAGACAAGGAGGAGTTATAGACGGTCCGGCATAGG R:TTGAAGACAAGGTGTCGATCGGACGTATTGTCCAAG |
| 5 | p*40S* B | F:TTGAAGACAACACCAAAGCAATCTGGTACATCACCC R:TTGAAGACAAGGTATCCTCCACAGACTCCTTGAGCC |
| 6 | p*40S* C | F:TTGAAGACAATACCTGATATAAGAATTGAGAGTTATACTCCGG R:TTGAAGACAACATTGTTTGCTGTCTATGTGGGGGACTG |
| 7 | QF DBD | F:TTGAAGACATAATGCCACCCAAGCGCAAAAC R:TTGAAGACAAACCTGAGGAGGCGGGTAATGCTCTTATTG |
| 8 | VP16 AD | F:TTGAAGACAAAGGTTTGAAAGCGGCGGGCCGG R:TTGAAGACAACGAACCCGGGGAGCATGTCAAGG |
| 9 | QF AD | F:TTGAAGACAAAGGTTTCGTCAGTTGGAGCTCCCTCCTA R:TTGAAGACAACGAACTGTTCGTATGTATTAATGTCGGAGA |
| 10 | *eGFP*-NLS | F:TTGAAGACAATTCGTTATGGTGAGCAAGGGCGAGGAGC R:TTGAAGACAAAAGCTTAGACCTTCCGCTTCTTCTTTGGCTTGTACAGCTCGTCCATGCC |
| 11 | t*tif35* | F:ACAGCGGAAGACAAGCTTACTTCTTTATCGGTTCTCTCTTACGAC R:ACAGCGGAAGACAAAGCGGTGCTTGGGATGTTCCATGGTAGCTGTG |
| 12 | p*gpdA* | F:ACAGCGGAAGACAAGGAGTGGATCCCCCGGGCTGCAGG R:GAAGACTTCATTGTGATGTCTGCTCAAGCGGG |
| 13 | *amdS;* A | F:GAAGACTTAATGCCTCAATCCTGGGAAGAAC R:TGAAGACAAGTCATCCGCAGGCAGCGTCTG |
| 14 | *amdS:* B | F:TGAAGACAATGACAGCGTTATTGATTTCCCAAAGAAATCG R:TGAAGACAAGGTGTCTTGTGCTTTGCGTAGTATTCA |
| 15 | *amdS;* C | F:TGAAGACAACACCCGTTGGTCCACTCCAT R:TGAAGACAAGAAAACGGCACCGGCTTTGCGG |
| 16 | *amdS;* D | F:TGAAGACAATTTCTACGTCAAGACCTCTGTCCCG R:ACAGCGGAAGACAACATTACTTCATCAGTGACTGCCCGTCTCGTATATAGTATAAAA |
| 17 | *amdS;* E | F:TTGAAGACAAAATGTTAGACCTCCGCCTCTTCAC R:TTGAAGACAAGGTATGACCGGCTTTGGCGAGTGCG |
| 18 | *amdS;* F | F:TTGAAGACAATACCGTGACCCCGTGGACGC R:GAAGACAAAAGCCTATGGAGTCACCACATTTCC |
| 19 | t*amdS* | F:ACAGCGGAAGACAAGCTTCTAATAAGTGTCAGATAGCAAT R:ACAGCGGAAGACAAAGCGTACCGCTCGTACCATGGGTT |
| 20 | p*pcbC;* A | F:ACAGCGGAAGACAAGGAGCTGCATTGGTCTGCCATTGCAG R:TGAAGACAAAGATTAGTAAGTACTTATCATTACCGTG |
| 21 | p*pcbC;* B | F:TGAAGACAAATCTTCGAGCGGGGGAGTGTT R:ACAGCGGAAGACAACATTGGTGTCTAGAAAAATAATGGTGAA |
| 22 | p*pcbAB;* A | F:TTGAAGACAAGGAGGTGCCTTACTGGATGGGGCC R:TTGAAGACAAAGTGCTTCGAGCGGGGGAGTG |
| 23 | p*pcbAB;* B | F:TTGAAGACAACACTAGTAAGTACTTATCATTACCGTGCCAG R:TTGAAGACAACATTGTCTGTCAATGACCAATAATTGGTAGGG |
| 24 | *Pc*_pcbAB CP | F:TTGAAGACAACCATATCTTGTCTGCGGGCAGTG R:TTGAAGACAACATTGTCTGTCAATGACCAATAATTGGTAGGG |
| 25 | *Pc*_pcbAB CP ctrl | F:TTGAAGACAAGGAGATCTTGTCTGCGGGCAGTG R:TTGAAGACAACATTGTCTGTCAATGACCAATAATTGGTAGGG |
| 26 | *Sc*_ura3 CP | F:TTGAAGACAACCATCAGAAGGAAGAACGAAGGAAGGAG R:TTGAAGACAACATTGATTTATCTTCGTTTCCTGCAGGTTTTTG |
| 27 | *Pc*_pcbC CP | F:TTGAAGACAACCATCGTATAATGTCTCCAGGTTGTCTCAGC R:TTGAAGACAACATTGGTGTCTAGAAAAATAATGGTGAAAACTTG |
| 28 | *Sc*_ura3 CP ctrl | F:TTGAAGACAAGGAGCAGAAGGAAGAACGAAGGAAGGA R:TTGAAGACAACATTGATTTATCTTCGTTTCCTGCAGGTTTTTG |
| 29 | *Pc*_pcbC CP ctrl | F:TTGAAGACAAGGAGCGTATAATGTCTCCAGGTTGTCTCAGC R:TTGAAGACAACATTGGTGTCTAGAAAAATAATGGTGAAAACTTG |
| 30 | *Pc*_phl CP | F:TTGAAGACAACCATCGTCACTTGTCATAAATCTCCCCTCA R:TTGAAGACAACATTTTTCCCAAATCCTCGGGGTAATATAGGG |
| 31 | *Pc*_phl CP ctrl | F:TTGAAGACAAGGAGCGTCACTTGTCATAAATCTCCCCTCA R:TTGAAGACAACATTTTTCCCAAATCCTCGGGGTAATATAGGG |
| 32 | *Pc*_penDE CP | F:TTGAAGACAACCATAGACTAGGCGGATGCAGCA R:TTGAAGACAACATTTTCTGCTGCGGGTCGGAAG |
| 33 | *Pc*_penDE CP ctrl | F:TTGAAGACAAGGAGAGACTAGGCGGATGCAGCA R:TTGAAGACAACATTTTCTGCTGCGGGTCGGAAG |
| 34 | *DsRe*d-SKL; A | F:TTGAAGACAAAATGGCCTCCTCCGAGGACGTCA R:TGAAGACAAAGTTTTCTTCTGCATTACGGGGCC |
| 35 | *DsRed*-SKL; B | F:TGAAGACAAAACTATGGGCTGGGAGGCCT R:TTGAAGACAAAAGCTTACAGCTTCGACTTGTACAATTCG |
| 36 | t*act1* | F:ACAGCGGAAGACAAGCTTGTGCTTCTAAGGTATGAGTCGCA R:ACAGCGGAAGACAAAGCGCGCAGGGTTTGAGAACTCCGATCT |
| 37 | Pen3'flank; A | F:ACAGCGGAAGACAAGGAGACTTTAGACATACCTCTCGT R:TGAAGACAAGAATACGTCATACTTATTCTCTGA |
| 38 | Pen3'flank: B | F:TGAAGACATATTCTTGGCAATGTTTAAGCTTG R:ACAGCGGAAGACAAAGCGCCTGCAGGCTATCCGATATGCCGTCTGC |
| 39 | *Pc*_*pcbC* CP with *pcbC;* A | F:TTGGTCTCAGGAGTAGACCTGGCTGACGGAGA R:TTGGTCTCACTCCCGTATAATGTCTCCAGGTTGTCTCAGC |
| 40 | p*pcbAB* with *pcbAB* flank; A | F:TTGGTCTCACCATGCTTGCAGCCCAGATGCTTAC  R:TTGGTCTCAAGCGCCAGACTCGTGTCCTTACGGGTCGAC |
| 41 | *Pc*_pcbC CP with *pcbC*; B | F:TTGGTCTCAGGAGCAGCTCACTACCACGCAAATCT R:TTGGTCTCACTCCCGTATAATGTCTCCAGGTTGTCTCAGC |
| 42 | p*pcbAB* with *pcbAB* flank; B | F:TTGGTCTCACCATGCTTGCAGCCCAGATGCTTAC  R:TTGGTCTCAAGCGGACAGTCGGAGAAACGCAGAG |
| 43 | p*pcbAB* with *pcbAB* flank; C | F:TTGGTCTCACCATATCTTGTCTGCGGGCAGTG R:TTGGTCTCAAGCGGACAGTCGGAGAAACGCAGAG |
| 44 | 5'flank *penDE;* A | F:TTGGTCTCAAGCGTGCCGAGGAGCTGGATTGAG R:TTGGTCTCAATCCCAAATCCGAGGGTAATGCAG |
| 45 | 5'flank *penDE;* B | F:AAGGTCTCAGGATGACAGTGTAATCCGCCGCAAG R:TTGGTCTCAGGAGGACTGAACCTCTTCGAGATAACAAGATTTTTC |
| 46 | 3'flank *penDE;* A | F:TTGGTCTCAAATGCTTCACATCCTCTGTCAAGG R:TTGGTCTCACGTTTTCTTCGTTTTCCCTCGGATGAGATC |
| 47 | 3'flank *penDE;* B | F:TTGGTCTCAAACGGACGAAGAGCTTAAACAGG R:TTGGTCTCAAGCGGACCCTGAAGGTGAAGGGC |

Table S4: MoClo plasmid construction. Plasmids were constructed using PCR products, synthetic DNA fragments and MoClo compatible plasmids. Description of DNA parts cloned into MoClo vectors listed under Part IDs, described in Table S2-3.

| **Plasmid** | **Description** | **Part IDs** | **MoClo vector** |
| --- | --- | --- | --- |
| pZB0_1 | 5' flanking region for integration at the deleted penicillin cluster of DS68530 | 1, 2, 3 | pICH41331 |
| pZB0_21 | promoter of AN0465; *40S* ribosomal protein S8e | 4, 5, 6 | pICH41295 |
| pFG0_1 | promoter of An11g02040; *gndA* | A | pICH41295 |
| pLM0_1 | DNA binding domain of QF TF | 7 | pICH41258 |
| pLM0_5 | Activator domain of VP16 | 8 | pAGM1299 |
| pLM0_2 | Activator domain of QF TF | 9 | pAGM1299 |
| pYN0_29 | *eGFP-NLS* | 10 | pAGM1301 |
| pYN0_10 | terminator of PC22g19890; *TIF35* | 11 | pICH41276 |
| pLM0_12 | promoter of AN8041; *gpdA* | 12 | pICH41295 |
| pLM0_11 | AN8777; *amdS* | 13, 14, 15, 16, 17, 18 | pICH41308 |
| pZB0_20 | terminator of AN8777; *amdS* | 19 | pICH41276 |
| pYN0_58 | 1xQUAS | B | pAGM1251 |
| pLM0_3 | 5xQUAS | C | pAGM1251 |
| pLM0_8 | 11xQUAS | G | pAGM1251 |
| pZB_0_23 | full length promoter of Pc21g21380; *pcbC* | 20, 21 | pICH41295 |
| pYN0_85 | full length promoter of Pc21g21390; *pcbAB* | 22, 23 | pICH47761 |
| pYN0_63 | CP of Pc21g21390; *pcbAB* | 24 | pAGM1276 |
| pYN0_64 | CP of Pc21g21390; *pcbAB* (No QUAS control) | 25 | pICH41295 |
| pYN0_21 | CP of YEL021W; *URA3* | 26 | pAGM1276 |
| pYN0_22 | CP of AN0098; *nirA* | H | pAGM1276 |
| pYN0_23 | CP of Pc21g21380; *pcbC* | 27 | pAGM1276 |
| pYN0_24 | CP of YEL021W; *URA3* (No QUAS control) | 28 | pICH41295 |
| pYN0_25 | CP of AN0098; *nirA* (No QUAS control) | I | pICH41295 |
| pYN0_26 | CP of Pc21g2138; *pcbC* (No QUAS control) | 29 | pICH41295 |
| pYN0_67 | CP of Pc22g14900; *phl* | 30 | pAGM1276 |
| pYN0_68 | CP of Pc22g14900; *phl* (No QUAS control) | 31 | pICH41295 |
| pYN0_65 | CP of Pc21g21370; *PenDE* | 32 | pAGM1276 |
| pYN0_66 | CP of Pc21g21370; *PenDE* (No QUAS control) | 33 | pAGM1251 |
| pZB0_26 | *DsRed-SKL* | 34, 35 | pICH41308 |
| pYN0_9 | terminator of AN6542; *Act1* | 36 | pICH41276 |
| pZB0_2 | 3' flanking region for integration at the deleted penicillin cluster of DS68530 | 37, 38 | pICH41331 |
| pYN1_77 | marker free donor DNA for 5xQUAS regulated CP of *PcbC* and p*pcbAB,* for making Strain 25 | pLM0_3, 39, 40 | pICH47761 |
| pYN1_80 | marker free donor DNA for 11xQUAS regulated CP of *PcbC* and p*pcbAB,* for making Strain 26 | pLM0_8, 41, 42 | pICH47761 |
| pYN1_81 | marker free donor DNA for 5xQUAS regulated CP of *PcbC* and CP of *pcbAB,* for making Strain 23 | pLM0_3, 41, 42 | pICH47761 |
| pYN1_82 | marker free donor DNA for 11xQUAS regulated CP of *PcbC* and CP of *pcbAB,* for making Strain 24 | pLM0_8, 41, 43 | pICH47761 |
| pYN1_78 | 5' flanking region for integration at *PenDE* of DS68530 | 44, 45 | pICH47732 |
| pYN1_79 | 3' flanking region for integration at *penDE* of DS68530 | pLM0_5,  pYN0_65, 46, 47 | pICH47761 |
| pYN2_71 | *amdS* marker containing donor DNA for integrating the STF and the 5xQUAS regulated CP of *PenDE* at *penDE* of DS68530 | pYN1_78, pLM1_52, pYN1_65,  pYN1_79,  pICH41780 | pAGM4673 |

Table S5: Primers used for qPCR

| **Target** | **Primers** |
| --- | --- |
| γ-actin | F:TTCTTGGCCTCGAGTCTGGCGG R:GTGATCTCCTTCTGCATACGGTCG |
| *pcbC* | F:AGGGTTACCTCGATATCGAGGCG R:GTCGCCGTACGAGATTGGCCG |
| *penDE* | F:CATCCTCTGTCAAGGCACTCC R:CCATCTTTCCTCGATCACGC |
| STF | F:TATATCATGGCCGACAAGCA R:GAACTCCAGCAGGACCATGT |

**REFERENCES**

1. Kaplan N, Moore IK, Fondufe-Mittendorf Y, Gossett AJ, Tillo D, Field Y, LeProust EM, Hughes, TR, Lieb JD, Widom J, Segal E. The DNA-encoded nucleosome organization of a eukaryotic genome. Nature. 2009;458: 362-366.

2. Raveh-Sadka T, Levo M, Shabi U, Shany B, Keren L, Lotan-Pompan M, Zeevi D, Sharon E, Weinberger A, Segal E. Manipulating nucleosome disfavoring sequences allows fine-tune regulation of gene expression in yeast. Nat Genet. 2012;44: 743-750.

3. Šmidák R, Jopčík M, Kralovičová M, Gajdošíková J, Kormanec J, Timko J, Turňa J. Core promoters of the penicillin biosynthesis genes and quantitative RT-PCR analysis of these genes in high and low production strain of *Penicillium chrysogenum*. Folia Microbiol. 2010;55:126-132.

4. Losson R, Fuchs RPP, Lacroute F. Yeast promoters URA1 and URA3: Examples of positive control. J Mol Biol. 1985;185:65-81.
